# Underwater Networked Wireless Sensor Data Collection for Computational Intelligence Techniques: Issues, Challenges, and Approaches

**DOI:** 10.1109/ACCESS.2020.3007502

**Published:** 2020-07-06

**Authors:** Osho Gupta, Nitin Goyal, Divya Anand, Seifedine Kadry, Yunyoung Nam, Aman Singh

**Affiliations:** 1 Chitkara University Institute of Engineering and Technology, Chitkara UniversityPunjabIndia; 2 Department of Computer Science and EngineeringLovely Professional University 126208 Phagwara144411India; 3 Department of Mathematics and Computer ScienceFaculty of ScienceBeirut Arab University 67025 Beirut11072809Lebanon; 4 Department of Computer Science and EngineeringSoonchunhyang University 37969 Asan31538South Korea

**Keywords:** Acoustic sensor network, coronavirus (COVID-19), computational intelligence, routing, underwater sensor network

## Abstract

Underwater wireless sensor networks (UWSNs) is emerging as an advance terminology for monitoring and controlling the underwater aquatic life. This technology determines the undiscovered resources present in the water through computational intelligence (CI) techniques. CI here pertains to the capability of a system to acquire a specific task from data or experimental surveillance below the water. In today’s time data is considered as the identity for everything that exists in nature, whether that data is related to human beings, machines or any type of device like internet of underwater things (IoUT). The collected data should be correct, complete and fulfill the requirements of a particular task to be done. Underwater data collection is very tough because of sensors mobility due to water drift 3 meters/sec, crest and trough. A lot of packet drop also exists due to underwater conditions that hurdles the data collection process. Various techniques already exists for efficient collection of data below the water but these are not properly classified. This manuscript has summarized the concept of data collection in UWSN along with its classification based on routing. Also, a short discussion about existence of CORONA below the water along with water purification is carried out. Furthermore, some data routing approaches are also analyzed on the basis of quality of service parameters and the current challenges to be tackled during data collection are also discussed.

## I. Introduction

Our earth has been covered with around 70 percent of the water which can be considered as the most valuable resources present on the earth. Around millions of living species of the aquatic life depends on the ocean water as it is the main habitat of the living organisms present on the earth [1]. Earth water absorb around one-fourth of the carbon-dioxide generated by the human activities. Transportation is one of the basic medium served by the water which human beings are using a lot. Despite all this it is known that around 90 percent of the oceanic part cannot be seen with the naked eyes or it is unreachable, still there are thousands of resources which need be discover in this giant ocean. It is very important to understand the natural resources that have to be developing deep under the water for the development of the country. With this it has been a great chance to more understand the mother earth when more and more research is done within the water. Moreover there are many current technologies going on but the major drawback among all these technologies are that they are not cost effective it is found to be expensive and proven as not adaptable. There are many devices which are generally used for the communication within the water but still these devices have some drawbacks or they are lagging behind with some type of important features [2]. With the lack of devices which helps to communicate or transfer data under the water there are many things that still come to know like tracking of the device, configuration of the device and collection of the data. For this the most efficient and reliable technology has overcome all the existing technology that is Underwater Wireless Sensor network (UWSN). UWSN is the most advanced and efficient network that has been used nowadays [3]. For millions of users and the devices they are using UWSN plays a very important and the significant role in providing services to the people and also transmission of the data [4]. In UWSN various sensor nodes are arrayed and stay connected with neighboring nodes such that they will be able to send and receive data under the sea [5]. These sensors after collecting the data send it to the sink node. Sink nodes are the processing system of the UWSN after collecting all the information by the sensor nodes this information is transferred to the sink nodes, sink nodes preprocess the data [6]. For the collection of data in water acoustic modems have been used, these modems are capable to understand commands and collects all the retrieve data which has been send immediately to the monitoring center [7]. Still acoustic modems facing many problems during transmission under the water these modems have been suffering from low bandwidth, loss of path at higher levels, high volume of noise, irregular delays and multi path propagation of the light deep below the water. All the above issues create challenges for the data collection in UWSN. The authors have compared various stimulating issues of computational intelligence approaches that degrade the performance of the collecting data in UWSN and provide various methods to overcome with this problem in a better manner. The main contributions of this manuscript are summarized below:

a) We survey data collection approaches and classifying them on the basis of routing.
b) We also presented comparative analysis of some of the existing state-of-the-art data routing techniques based on simulation.
c) We describe in detail, the overall data collection process covering all these schemes w.r.t. data discovery, data forwarding, data delivery and node mobility.
d) We comprehensively discuss the pandemic COVID-19 in oceans, rivers etc. with an existing water filtration model of Dubai.
e) We describe the upcoming challenges faced by the data collection approaches in underwater wireless sensor network.

## II. Related Work

UWSN is also known as acoustic sensor networks (ASNs). Computational intelligence is said to be the set of nature-inspired computational methodologies to notify complex real-world problems like underwater monitoring and data collection where mathematical or traditional models can be useless. Some efficient techniques and algorithms have been proposed for the purpose of adequate data collection. Similarly some techniques are still under testing for data collection purpose.

Rahman *et al.* [8] projected a technique for the delay tolerant networks (DTN) where many efforts have been done in developing the architecture and algorithms for data collection in UWSN, which can be characterized by the delays for the long message and some frequent partitions. To solve that problem an enhanced version of the model has been developed in which current state can be known for the DTN and also various adaptive routing protocols have been proposed for the UWSN. Bouk *et al.* [9] proposed a delay tolerant data-dolphin scheme have been proposed for the energy efficient in UWSN. Mobile elements which can be easily controlled or can be controllable are proposed for the efficient transmission of data in the underwater environment. Janardanan *et al.* [10] discussed scheduling based on mobile element in the sensor network of terrestrial region for the collection of data where mobile sinks based meeting approach is planned and undergoes proper investigation. In this scheduling approach, it is proved that even if a network consist of mobile nodes the DTN approach is found to be very effective for successful delivery of data. Hence various analytical techniques have been implemented to check the impact on the performance of the network. In this work a sizably voluminous body is designed and built with submersed robots and sensor networks is presented. Ample of the submersed work till now is with cabled connections requiring consequential engineering, acoustic networking, and maintenance issues.

Kilfoyle *et al.* [11] provides an excellent review of submerged acoustic communications. Some challenges while designing submersed sensor networks is depicted in this manuscript. With the demand of Robots utilization and sensor network all the ranging and the communication in an optical system is clearly mentioned. Data muling delivers an efficient way to increase coverage of the network and reduces the consumption of the power in a sensor based network system [12]. In Jain *et al.* [13] proposed a method where mules related to mobile can be used such as animals and buses which help in amassing of data in a sensor network which is sparsely deployed by across in this network. Enabling of communication can be done only when there is a close proximity between the mobile mules and the sensors. Potency consumption can be decreased when the data is transmitted over shorter distances. Aziz *et al.* [14] expresses about various infrastructure of the network, hardware used, and analysis which can be done experimentally with data retrieval and collection of data. They reported on a prototype developed, built, and used for an underwater sensor network. Tripathi *et al.* [15] demonstrated in their work that in underwater network based on sensors are feasible in nature and at that time muling of data gives an effective way to store, and collect data for a long period of time. With the introduction of the multihop routing concept muling of data gives a huge advantage over a network communication based on the acoustic links. For the collection of data mainly three schemes can be implemented or used in UWSNs. Scheme of multi-hop accumulation can be used for the prediction of the various methods of the data accumulation this has to be done because the mobile edge computing performance is circumscribed. For the accumulation of the data AUV-availed schemes have been used this only happen because of development in mobile edge systems. Recently, data accumulation adopts a system that changed multi-hop structure with AUV-availed data accumulation structure. In the multi-hop scheme of data accumulation, data is amassed by the source node and the relay node is culled in such a way that data is forwarded to the sink node. But in this scheme of multi-hop, problem of energy consumption cannot be solved in UWSNs, so the scheme of data accumulation which involves AUV is proposed. For 3D UWSN having kenned deployment information, AUV simply needs to build a least probability neighborhood coverage set for data latency [16]. To reduce the problem of packets delay in accumulation scheme of AUV many researchers mixed it with data accumulation scheme of multi-hop. In the coalescence scheme, all the monitoring areas and the nodes are not traversed by the AUV. In the clustering network which comprises of multiple cluster heads (CHs) gateway nodes is responsible for the forwarding of the data packet to the CH. Hence, for the accumulation of data only the gateway nodes and the CHs are visited by the AUV [17].

In this section a brief survey of existing data collection techniques for UWSNs was presented. The overall novel classification of data collection techniques is shown in next section 3. Furthermore, the various limitations of existing solutions along with its benefits are also summarized with the help of table in next section.

## III. Data Collection in UWSN

Early detection of activities like underwater submarine attack, oil leakages in big plants of mine mining, assisted navigation to avoid ship wreckage, tectonic plates movement generating tsunami and high tides, exact pollution sources areas in water motivate authors and researchers to work for data collection. It’s been a challenging task for the efficient collection of data in UWSN because of many undersea problems that cannot be directly rendered into binary language (values of 0 and 1) for system processing. Hence computational intelligence approaches provides solutions. This may happen because of changing characteristics of underwater environment or the acoustic system [18]–[23]. Some of the characteristics to make a tough task easier for the collection of data deep into the water have been discussed as follows:

a) There are very few chances of using static topology in UWSN because most of the topologies are dynamic in nature as there are regular deviations in the network topology of the UWSN. This regular change happens because of the involuntary mobility nature of the sensor nodes caused of ocean currents and uncertainty in the link of the sensor nodes. Various traditional routing protocols such as reactive or proactive proven to be impractical for the transmission of the data.
b) Aquatic environment put a great effect on the acoustic link below the water, signal generated by the acoustic links are fully absorbed or get affected as the frequency increases in the water. Sometimes it also get disturb from natural and man-made activities perform on the water like generation of natural waves, waves develop by submarines and turbulence generated by the moving vehicles on the water. As the signal gets reflected into different directions so it produces the multipath propagation effect.
c) In UWSN data has been delivered via thousands of acoustic links. These links are very effective and sensitive in nature. Many natural parameters of the water like pressure, density, and volume and oceans currents can effects the data transferring within these links. Sometimes these links are not able to send complete information to the monitoring center due to bad weather condition, temperature conditions and increase in salinity levels of the water. Multiple path effect in the water also rendered the successful delivery of data.
d) Due to the covetous nature of UWSN for energy, lot of energy is required for the transmission of data such that the cost for each transmission is very high that required dozens of watts for the transmission of data, hence acoustic modems are not reliable for transferring the data. During the time of fault in UWSN lot of cost is required due to high range of battery prices used in UWSN and bigger cost for the ship management team which repairs the fault area underwater.

**FIGURE 1.**
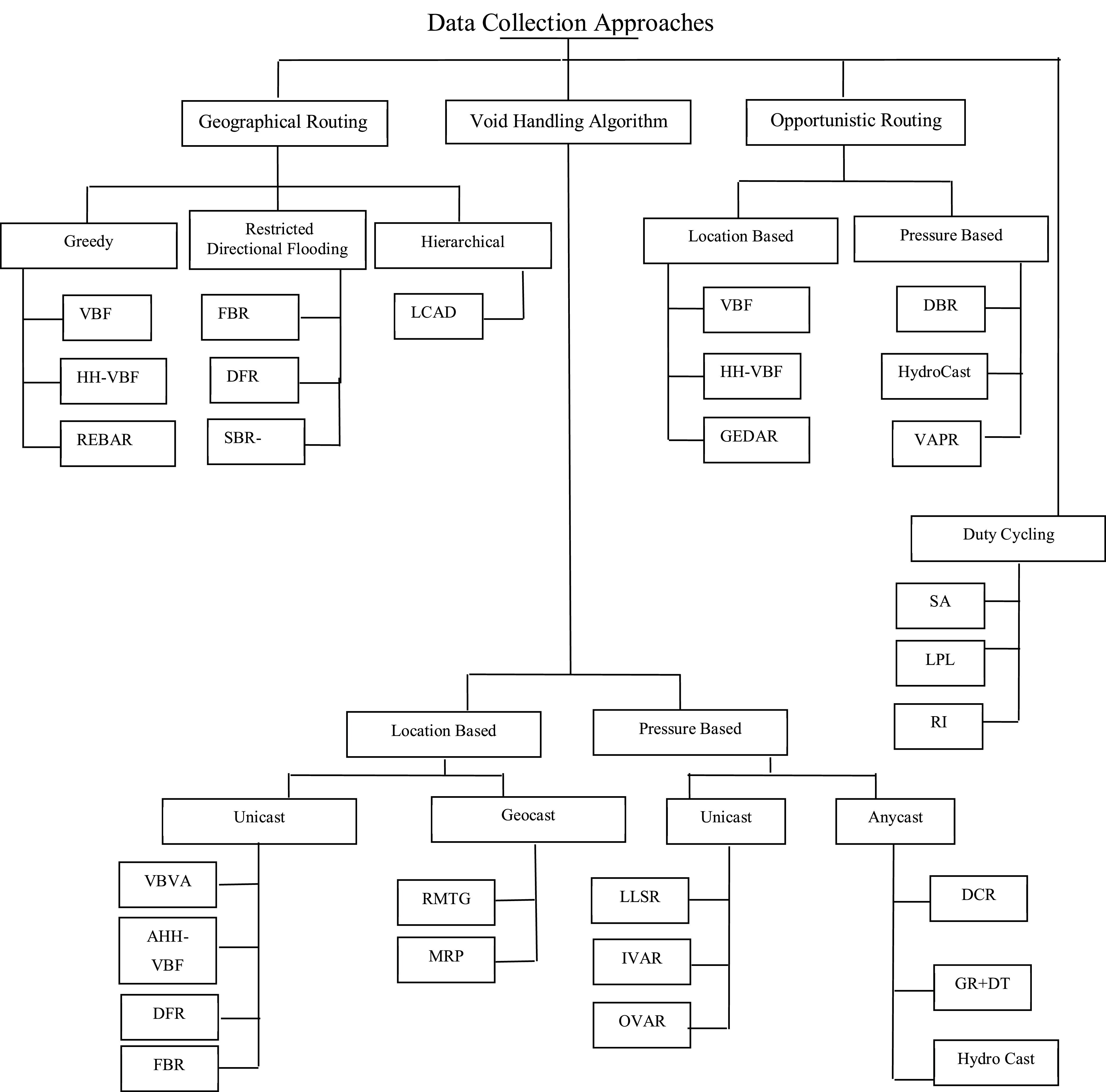
Classification of efficient data collection approaches in UWSN.

Since it seems that there is imbalance in energy consumption at the time of the transmission, for that there must me one central or parent nodes in UWSN which restricts the network traffic to pass only the needed data [24]–[26]. Being having control of all other nodes the battery life of the central node will be depleted sooner as the central node is responsible for the transmission of data at the time of battery faults the node is unable to transmit the data results in the error in working of the application [27]–[29]. So not only the energy efficient property of the UWSN is required it is also be balanced power supplier to all other nodes of underwater network [30]–[32]. All the data collection approaches of UWSN can be classified on the basis of routing strategy and shown in figure 1:

### A. Data Collection Classification

Data collection approaches and techniques in UWSN are classified mainly into four groups as geographical routing, void handling algorithms, opportunistic routing, and duty cycling based approaches. These are briefly discussed as below:

#### 1) Geographical Routing

Geographical Routing is the most simple, scalable and efficient protocol which generally works on wireless sensor network. This protocol does not uses for any type of discovery process in the water it is for end-to end location of the connecting path. This routing protocol uses end-to-end location of the nearest nodes. When one node got a data packet it gradually sends that packet to the destination node which is close to the sending node among all other nodes. Once the closest or the nearest node is found it sends data to that node. Still geographic routing suffers from the major drawback which can be called as void region of communication. Void region is a region when the current sender node is unable to find the closest node where to send the packet. All the nodes in the void region are known as void node. Whenever this type of condition arises, this protocol tries to recover the process of transmission if still not achievable the data packet has been discarded from the queue resulting in the loss of data [33]–[39]. Geographical routing approaches can be divided mainly on 3 basics define as follows:

##### a: Greedy Approaches

These approaches do not maintain and create paths from source to the destination, by which source node contains information about the distance up to the receiving node where packet has to be transferred. It utilizes the optimization process for selecting the next hop. Some of the protocols based on this approach are:

VBF (vector based forwarding): It’s a full localization protocol based on AOA (angel of arrival) technique to determine the position of the node and signal strength, information of the sender location, all is carried in the packet. AOA technique is used to calculate the position when the node receives the packet, if the packet is in the pipe determine by the node, packet transmission takes place if packet is not there it will be discarded.
HH-VBF (hop-by-hop VBF): As in case of VBF from source to the sink unique virtual pipe is created but in case of HH-VBF virtual pipe is created at each hop that’s why hop-by-hop approach is used for routing. When the packet was received the node compares the vector from the sender towards the sink and calculates distance to that vector.
REBAR (reliable and energy balanced algorithm): It’s a routing protocol based on the location which focus on the three main problems to tackle in UWSN. First to analyze the consumption of energy by the nodes REBAR uses sphere energy depletion model. Another thing is the node mobility factor which balances the energy depletion in the network.

##### b: Restricted Directional Flooding Approaches

In restricted directional flooding packets will be broadcasted by the sender to all the neighbors with single hop towards the destination. The packet is then checked by the receiving node whether it is the part of the given set of nodes. If yes then packet retransmission takes place otherwise packet will be dropped. Some of the protocols based on this approach are:

FBR (focused beam routing): It is a routing protocol based on location and is efficient in energy. In FBR nodes in the network knows only its own location and the location of the final destination node. In FBR forwarding of data packet is done by variables levels of power transmission.
DFR (directional flooding based routing protocol): Aquatic environment conditions and high mobility of nodes are the factors that lead to the loss of packet during the time of routing, so DFR mainly focus on this thing on how to decrease the loss of packets at such a harsh condition.
SBR-DLP (sector-based routing having destination location prediction): In this every node in the network recognizes its personal locality but the prediction has to be done for the destination node location. It reduces the need for accurate knowledge in determining the location of the destination node.

##### c: Hierarchical Approaches

LCAD (location-based clustering algorithm for data gathering): It’s a geographical location based clustering algorithm, in which network is divided into 3-D grids. A single cluster is comprised in a single grid. Communication of data is divided into three phases:

(I) Phase of set-up, in which selection of CH exists
(II) phase of data gathering, in which transmission of data is done from nodes to CH.
(III) phase of transmission, in which data collected by the CHs is transmitted to the base station (BS).

#### 2) Void Handling Algorithms

These Algorithms are mostly used or implemented in various geo-routing protocols. These algorithms are responsible for providing route to the data packets which are present in the void region or void nodes [36], [40]–[50]. By knowing three procedures void handling algorithms have been implemented when the

a) Methodologies for the void region have been passed.
b) Methodology based on power control
c) Methodology based on assisted mobility.

The first methodology that is bypass the void region makes route to data packets by avoiding the void region. In this process the node present in the void region is connected to some other greedy node which is empty or is capable of holding data packets which can easily continue the transmission process of the data packets. By using this scheme the main improvement in UWSN network is that it doesn’t affect the topology of network and nodes location. But due to this energy consumption can be increased because of discovery of path and its maintenance.

The seconds methodology is power control based this is quite simple and scalable. The best thing of this method is that it works on without making or discovers a node in the void region; it simply increases the range of communication of the sender node such that it can easily find the nearest destination node.it also increases the energy consumption.

The third methodology is mobility based it is very energy efficient and less power consumption technique used in the UWSN network. This method allows the sending void node to move to another location outside the void region where it can easily find the nearest destination node so that it can easily transmits data packets as soon as possible without delaying time and energy.

Void handling approaches can be divided mainly on location base or pressure bases which are further divided on the basics of unicast or geocast and unicast or anycast respectively as follows:

##### a: Location Based

I. Unicast: It is the single sink architecture which is suitable for small networks, in unicast when single sink receive the packet it means packet delivery is successful. Determination of the void can be done with respect to the single sink. In unicast we always have limited number of data packet transferring paths. Some of the unicast protocols are given below:

VBVA (Vector-Based Void Avoidance): it’s a stateless, receiver based and reactive method that is planned to decrease the void communication bad effect in the vector-based routing like HH-VBF and VBF.
AHH-VBF (Adaptive Hop-by-Hop Vector-Based Forwarding): To prevent the void problems AHH-VBF technique can be used. The best thing about AHH-VBF is every node knows about the sender node, receiving node, sink location and itself.
DFR (Directional Flooding Routing): it is also a stateless, receiver based and location based routing scheme. To attain more reliability DFR takes advantage of controlled flooding approach in advanced with various link qualities.
FBR (Focused Beam Routing): In FBR sink node location, node of the sender and node itself is already known by each node. The main focus of FBR is to decrease consumption of energy by controlling the transmission power of the forwarding nodes.

II. Geocast: Here nodes are located at some geographical areas as per the destination. It is generally appropriate for bigger networks. The successful delivery of packet can be achieved when it is acknowledged by all other nodes in the geocasted region. Number of accessible path depends upon the covering area around the geocast region. Some of the geocast protocols are given below:

RMTG (Routing and Multicast Tree based Geocasting): It is the technique based on the 2D geocast in UWSN. It has whole detection ability of data distribution in an itemized geographical area with a collection of sensors. Each node in the RMTG distinguishes Destination area location, and neighboring nodes.
Mobicast routing protocol: it is an approach that used mobile geocasting that is having goal to collect information from the underwater zone even with water currents and various void areas.

##### b: Pressure Based

In this pressure gauge is used such that all nodes already aware of their depth. There is no need of any services based on the localization and also no knowledge required for the destination nodes location. Nodes in pressure based schemes sometimes gather depth information of the neighboring nodes. In these routing protocols are used only to gather the depth information. These techniques are further divided into different parts:

I. Unicast: it is the single sink architecture which is suitable for small networks, in unicast when single sink receive the packet it means packet delivery is successful. Determination of the void can be done with respect to the single sink.in unicast we always have limited number of data packet transferring paths:

LLSR (Location-free Link State Routing): Greedy hop-by-hop routing method is used by the LLSR by depend on some parameters like quality of the path, pressure and hop count. For the proximation of the node to the sink value of hop count is used such that LLSR can easily bypass the area of void in an easy way.
IVAR (Inherently Void Avoidance Routing): it provides the routing protocol which excludes all the path leading to the void area such that mode of recovery cannot be used. The transferring of the packet to the destination can only be done by determining the hop count and depth information of the each node.
OVAR (Opportunistic Void Avoidance Routing): To overcome the drawbacks of IVAR, OVAR routing is proposed. OVAR generally works on the beaconing procedure which is quite similar to IVAR to tackle the communication issue of the void areas. In OVAR candidate nodes are selected by the forwarding nodes and these nodes keep the ids of the candidate nodes in the header of the packet.

II. Anycast

DCR (Depth-Controlled Routing): It is the first routing protocol which is based on the geographic location this protocol generally uses control scheme network topology ta tackle void problems in UWSN. All the nodes in the DCR already know the sink location, nodes in the neighbors, and the pressure. With the help of the vertical movement in the nodes impact of void problem can be decreased in the network.
GR+DTC (Greedy Routing with Distributed Topology Control): A distributed algorithm has been proposed by the GRDTC in order to improve the robustness of the DCR. Nodes in the GRDTC know the sink location, pressure of the node and itself. All the nodes are able to determine the depth value from that it selects the void area.
HydroCast: It pressure and pressure of all the neighbors’ nodes is known by each node in the HydroCast, and distances of two hops in the neighbors. At the time of routing all the neighboring nodes subset is selected by the HydroCast with progress of maximum greedy to the destination considering problem of hidden terminal.

#### 3) Opportunistic Routing

In this approach the sender node in the void region select a candidate node which is responsible for setting up of priority levels to the other nodes. When the sender void node sends a data packet it is received by the candidate node, candidate checks the priority levels of all other connected nodes. The node with highest priority is able to receive data from the candidate node Further similarly for next transmission of the data packet candidate node again check the priority levels and transmits the data packet with highest priority node. With the decrease in the data transmission it automatically decreases the data collisions which can be a major advantage point for the UWSN data collection [51], [52]. Various protocols which come under opportunistic routing are as follows:

##### a: Location Based

VBF: It’s is a geographical routing protocol that needs full localization. AOA (angel of arrival) technique is used to determine the position of the node and signal strength, information of the sender location all is carried in the packet. When the packet was received by the node position is calculated with the help of the AOA technique, then the packet is determined by the node if it is present in the pipe, packet transmission can be continued else packet will be discarded.
HH-VBF (hop-by-hop VBF): As in case of VBF from source to the sink unique virtual pipe is created but in case of HH-VBF virtual pipe is created at each hop that’s why hop-by-hop approach is used for routing. When the packet was received the node compares the vector from the sender towards the sink and calculates distance to that vector.
GEDAR (Geographic Opportunistic Routing Depth Adjustment-Based Topology): In GEDAR information about location is combined with the opportunistic routing. The next forwarder is selected with the help of greedy forwarding when the intermediate node receives the data packet. Strategy of recovery is introduced whenever the hole of communication is encountered.

##### b: Pressure Based

DBR (Depth-Based Routing): In DBR candidate list of the data packets is generated by the sensor node depth information. In DBR each node of the network is attached with the pressure sensors. During the time of the transmission the sender node measures the depth and this information is attached with the header part of the data packet. Receiving node check the values of depth with the receiving values from the sensor nodes, if current node depth is less it can be taken as the forwarding node.
HydroCast (Hydraulic Pressure Based Anycast): All the communication links in the network are handled by the Hydrocast protocol to make this protocol better than DBR. Both the DBR and Hydrocast have similar working as for the generation of the candidate list and forwarding both uses the depth information.
VAPR (Void-Aware Pressure Routing): To tackle the holes of communication in the network VAPR protocol is proposed. To transmit the information regarding depth and nodes direction this protocol uses the beacon message in the network. After getting the nodes direction information all the potential void nodes is removed from the candidate list.

#### 4) Duty Cycling

For the conservation of energy basically in the networks that are based on energy constraint, approach of duty cycling can be implemented. Network based on duty-cycled where each node changes its state between sleep and active mode periodically. By preventing the energy waste because of sensor nodes operation of idle listening network with extended lifetime can be achieved that can be quite a dominating work in a load with low traffic of a UWSN network [53]–[55]. There are three main protocols that come under Duty Cycling:

Simple asynchronous (SA): In this node changes between sleep and active time periodically. When there is any information packet to send, it also sends the address to the next-hop node.
Low-power listening (LPL): When there is availability of information packet to send by the node, a preamble has been sent as long as sleep time of the next neighbor node. When the next-hop wakes up and receives the preamble transmission it remains in the awake condition to receive the data packet.
Receiver initiated (RI): When node wakes up it transmits a short beacon to tell neighborhood about its active status and stay active until to receive packets as much it can.

All the data collection classification approaches of UWSN are compared as shown in table 1 below:

**TABLE 1:** Classification of Efficient Data Collection Approaches in UWSN

s.No	Ref.	Method Name	Type	QoS Metrics Used	Proposed Method	Advantages	Disadvantages
1.	Xie et al. [33]	VBF (Vector Based Forwarding), 2006	Geographical Greedy	Node Position, Signal Strength, Sender Location	Optimization Process for Selecting Next hops. Localized and Distributed Self Adaptation Algorithm	Reduce Energy Consumption by Discarding the Low Benefit Packets	Time Complexity
2.	Xie et al. [34]	HH-VBF (Hop-By- Hop VBF), 2010	Geographical Greedy	Link Quality, Success Rate, Energy Efficiency, Error Probability	Virtual Pipe is Created at Each Hop	Time and Energy Efficient, Success Rate in Sparse Network is More	Time Consuming Process
3.	Chen et al. [35]	REBAR (Reliable And Energy Balanced Algorithm), 2008	Geographical Greedy	Energy Efficiency, Delivery Ratio, Active Sensor Nodes, Node Mobility	Uses Sphere Energy Depletion Model	Energy Efficiency, Deal with Routing Voids	Quite a Lengthy Process
4.	Jomet et al. [36]	FBR (Focused Beam Routing), 2008	Geographical Restricted Directional Flooding	Delivery Delay, Energy Efficiency	Variable Transmission Power Levels are Used in the Forwarding of Data Packets	Energy Efficient, Consider Both Static and Mobile Nodes, Dynamic Route Discovery is Minimal	Location is Must Required
5.	Hwang et al. [37]	DFR (Directional Flooding Based Routing Protocol), 2008	Geographical Restricted Directional Flooding	Communication Overhead, Packet Delivery Ratio, Delay	Packet Flooding Technique in Hop-by-Hop Manner, Also Address Void Problem	Successful Delivery Over One- Hop Links, Higher Reliability, Less Overhead	Void Problem is Still There When no Node is near to Sink, Impact of Predefined Values is Not Considered
6.	Chirdchoo et al. [38]	SBR-DLP (Sector- Based with Destination Location Prediction), 2009	Geographical Restricted Directional Flooding	Long Propagation Delay, Node Mobility, High Channel Error Rate, Low Data Rate	Multi-Sector Based Routing	Mobility of Nodes Considered, Supportive for Dynamic Networks where Nodes Join and Leave at Random	Placing Back of Sink Node at Starting Position is Not Realistic.
7.	Anupama et al. [39]	LCAD (Location-Based Clustering), 2008	Geographical Hierarchical	Lifetime of the Node, Cluster Size	Clustering Algorithm	Fast Communication	Time Consuming Process
8.	Xie et al. [40]	VBVA (Vector-Based Void Avoidance), 2009	Void Handling Location	Based on 3D and Mobile Void Problems	3D Vector-Shift and Back-Pressure Methods are used to Handle Voids	Without knowing Network Topology, Handle Mobile Void Routing Problem	Exchanging Beacons Imposes Overhead in Communication
9.	Yu etal. [41]	AHH-VBF (Adaptive Hop-hy-Hop Vector- Based Forwarding), 2015	Void Handling Location Unicast	End-to-End Delay and Energy Consumption, Data Delivery Ratio, and End-to-End Latency	Neighbour Node Distribution, Pipeline Radius and Transmission Power Adjustment	Adaptive, Energy-Efficient and Reliable	Lower Data Delivery Ratio, Too Sensitive to Routing Pipe Radius Threshold
10.	Shin et al. [42]	DFR (Directional Flooding-Based Routing), 2012	Void Handling Location Unicast	E2E reliability Packet Delivery Ratio, Communication Overhead, Average End-to-End Delay	Uses Controlled Flooding Approach with Various link Qualities by Allowing at Least One Node to Participate	Reliable, Consider Node Mobility	Increasing No. of Duplicate Packets and Wasting the Network Resources like Energy Consumption
11.	Montana et al. [43]	FBR (Focused Beam Routing), 2008	Void Handling Location Unicast	Average Energy Consumption, End-To-End Delay, Bandwidth Consumption	Control the Transmission Power as Because Sink Node and Sender Node Location is Already known by Each Node	Increased Lifetime, Multicast Operation is Performed at Low Power Level	If Sender does not Receive any Response then Power is Increased
12.	Dhurandher et al. [44]	RMTG (Routing and Multicast Tree Geocasting), 2010	Void Handling Location Geocast	Accuracy, Overhead, End-to-End Latency, and Packet Delivery	Uses Greedy Forwarding and Previous Hop Handshaking to Route the Packets Along with Presence of Nodes in Other Parts	Memory Saving, Node Mobility Handling, Less Overhead, Detection Ability	No Model or Practical Implementation
13.	Chen et al. [45]	Mobicast routing protocol, 2013	Void Handling Location Geocast	Delivery Rate, Power Consumption, Message Overhead, Average Delay, Throughput	Dynamically Estimate the Accurate 3- D by Apple Slice Technique to Build Multiple Segments to Surround the Hole and Assure Routing Path Continuity	Mobility is Considered, Overcome the Hole Problem	High Energy Consuming
14.	Barbeau et al. [46]	LLSR (Location-Free Link State Routing), 2015	Void Handling Pressure Unicast	Avg. Delay, Receiving Rate	Every Node Provide the Quality Ranking of Path, for the Proximation of the Node to Sink	Easily Bypass Void Area, Recovery Mode Even when Topology Changes, Loop Free	Location of the Nodes has to Predetermine, Doesn’t Perform Well when huge Nodes
15.	Ghoreyshi et al. [47]	IVAR (Inherently Void Avoidance Routing), 2015	Void Handling Pressure Unicast	Packet Delivery Ratio, Avg. Hop Count, Propagation Deviation	Excludes all the Path Leading to Void Area hy Periodic Beaconing and Pressure Gauge Embedding	Requires Only Hop Count and Depth Information, Less Overhead, Work both in Multi & Single Sink Model	Time Consuming, when no. of Nodes is More than 700 then Propagation Deviation Increase
16.	Ghoreyshi et al. [48]	OVAR (Opportunistic Void Avoidance Routing), 2016	Void Handling Pressure Unicast	Delivery Ratio, Energy Consumption, E2E Delay, Hop Count, Propagation Deviation	Uses Periodic Beaconing Followed by Adjacency Graph, Selects Forwarding Set hy Removing the Hidden Nodes Based on Energy-Reliability Constraints	Can route packets in any direction, reliable acoustic transmission	Network Energy Balancing is Required
17.	Coutinho et al. [49]	DCR (Depth-Controlled Routing), 2013	Void Handling Pressure Anycast	Packet Delivery Ratio, Energy Consumption, avg. E2E Delay	Depth Adjustment of Disconnected and Void Nodes Along with Greedy Forwarding Data Process	Reliable, Efficient Technique	Node Mobility is not Considered
18.	Coutinho et al. [50]	GR+DTC (Greedy Routing with Distributed Topology), 2015	Void Handling Pressure Anycast	Packet Delivery Ratio, Delay, Energy Consumption, Depth Adjustment	Determine the Depth Value to Select the Void Area	Derive Favourable Topologies by Depth Adjustment, Improve the Robustness	Exchanging Beacons Imposes Communication Overhead in Highly Dynamic Networks
19.	Lee et al. [51]	Hydrocast, 2010	Void Handling Pressure Anycast	Degree of Channel Utilization Delivery Delay	Pressure on Node and Neighbor Nodes is known & Utilized at the Time of Routing when Neighboring Nodes Subset is Selected to the Destination	Consider Problem of Hidden Terminal, Very Less co-Channel Interference	Dense Topology Imposes High Cost and can’t Eradicate Void Problem
20.	Yan et al. [52]	DBR (Depth-Based Routing), 2008	Opportunistic Pressure	E2E Delay, Packet Delivery Ratio, Depth Threshold, and Energy Consumption	Uses Greedy Approach to Deliver Packets to the Sinks at the Water Surface Based on Local Information of Sensor Nodes	Only Depth Information Required, Reduced Cost	Greedy Approach May Fail, only Simple Multiple Sinks Cases are Considered
21.	Noh et al. [53]	VAPR (Void-Aware Pressure Routing), 2012	Opportunistic Pressure	Energy Efficiency, Latency	Uses the Periodic Beaconing and Features Greedy Opportunistic Directional Forwarding for Packet Delivery	Easy Method for Data Transmission, Lower Recovery Frequency, Handle Node Mobility	Inappropriate to Handle Voids
22.	Feng et al., [54]	Simple Asynchroaous (SA), 2015	Duty Cycling Protocol	Node Density, Throughput, Energy Consumption.	Uses a selection of network coder node algorithm before data transmission	Low Energy Consumption and Higher Reliability	Quite a Lengthy Process
23.	Coutinho et al. [55]	Low Power Listening Protocol (LPL), 2015	Duty Cycling Protocol	Packet Delivery Ratio, End-To-End Delay, Energy Consumption.	In This Method the Transmission Node which Contains the Data Packets Sends a Preamble to notify the Neighboring Nodes	Reduce Energy Consumption by Discarding the Low Benefit Packets	High Power Consumption at Time of Transferring Large Preambles
24.	Sun et al., [56]	Receiver Initiated (RI), 2008	Duty Cycling Protocol	Average Packet Delivery Ratio, Average Latency, Average Duty Cycle	To Deal With Different Traffic Loads this uses Receiver Initiated for transmission	Power Efficient, High Performance, High Throughput Levels	Time Consuming Process

Further in section 4, working model for data collection in case of UWSN is shown where various sensors are placed to monitor the physical changes in the sensor radius.

## IV. Working Model

Data collection is a challenging process of measuring or gathering information, involving many issues of interest in an established systematic fashion that enables one to monitor or assess underwater resources.

**FIGURE 2.**
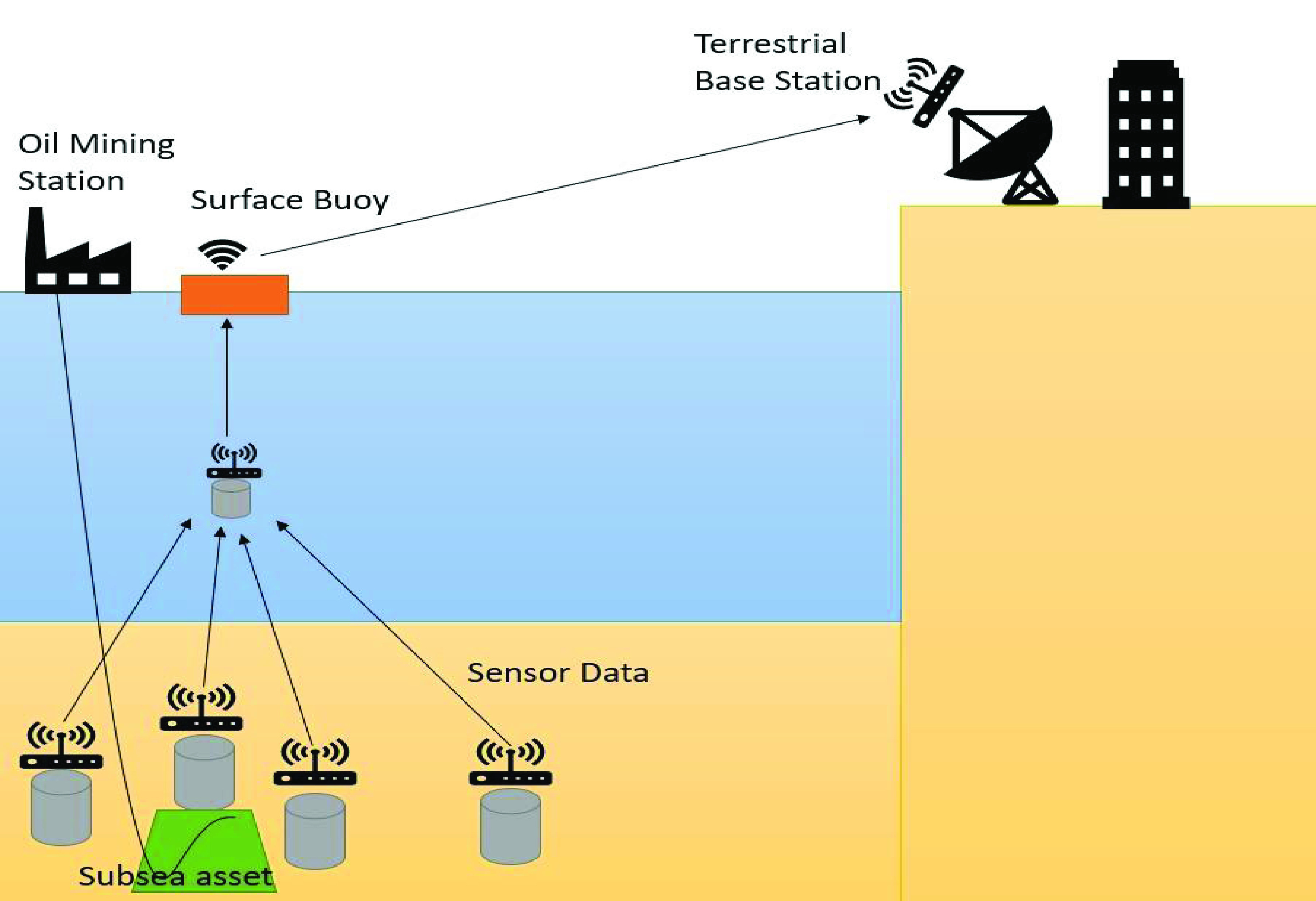
Data collection in underwater wireless sensor network.

The working and step-by-step process of data collection in UWSN is depicted in figure 2. In the figure there exists many devices that are deployed under the sea water have their unique kind of functioning. Some of the devices which have been used are sensor nodes, sink nodes, acoustic modems and BS. Sensor nodes are responsible for the extraction of data at deployed points. Acoustic modems or links are used to convert this data into signals from digital format that can easily travel into the water. Sink nodes play a very important role in this whole process as it helps to preprocess the data, the data which comes from the sensory nodes is sent to sink nodes which preprocess the data and extract valuable data and tells about what type of information the device is holding. Then the processed information is transferred to the terrestrial BS in the form of signals. Before reaching to the BS signal data is again converted into the digital data with the help of the acoustic modems. This digital data comes in the form of images or may be audio voices.

**FIGURE 3.**
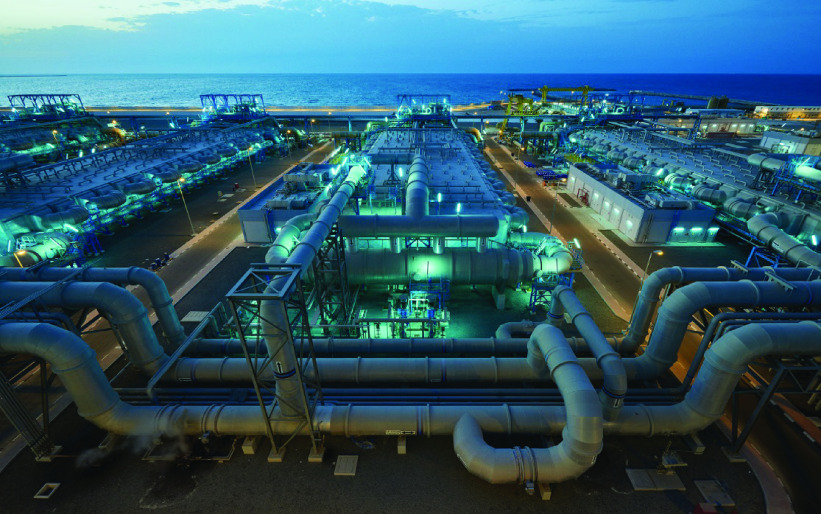
Dubai’s huge desalination plant [56].

It has been observed that there are very fewer chances to have COVID-19 in water but during this pandemic the use of masks, plastic and some other materials (may be non-biodegradable) in some cases can be thrown in sea water. If these materials are having virus on them then it can be spread to the sea animals. It is quite tough to collect data regarding infection or to monitor the health status of the underwater animals. But still in some of the viable animals like fish the implantable temperature sensors like DST Nano-T can be used for measuring body temperature. To monitor the infection in sea animals the only primary analyzing tool is temperature not behavior. Afterwards, data collection can take place by deploying node localization method if a particular sea animal shows the symptoms of COVID-19 or not. Further, if the water is to be used for drinking purpose then CORONA virus can be easily removed at the time of filtering of water. As per World Health Organization (WHO) a great initiative has been taken by Dubai as shown in figure 3, where large water purifiers are placed to purify gallons of water in a short time span. This technique is so helpful that the water purify by these purifiers are so pure making it fit for drinking also.

It has been suggested that in UWSN various sensors can be deployed under the water for data collection that check for temperature levels of the body of the sea animals. Any suspected animal found has to deliver an unconsciousness injection medicine with the help of the gun shot that has been taken for further testing. So it will help to reduce the spread of covid-19 in water [57].

## V. Experimental Analysis

For relative study in this manuscript, certain UWSN methods namely Intra and inter cluster communication (IICC) [58], Minimum average routing path clustering problem (MARPCP) [59] and Improved Data Aggregation for Cluster Based UWSNs (IDACB) [60] are considered. IICC is applicable to randomly deployed UWSNs where balancing the energy consumption of network and enhancing energy efficiency effectively during data aggregation is a challenge. MARPCP is used for intra-cluster communication. Through MARPCP, it is feasible to find the cluster heads in a given UWSN where the predicted hop distance from a node to its nearest underwater sink is minimized. IDACB reduces the data redundancy and ensure energy efficient collision-free transmission. This technique improves upon the results of the existing techniques in terms of energy consumption, end-to-end delay and packet delivery ratio. We executed these existing protocols on the network simulator NS 2.30. Authors have around 10 repetitive simulation rounds for every varying packet size and the average reading of these simulation rounds is considered as well as shown through graphs. The other simulation factors used are also summarized in Table 2.

**TABLE 2:** Simulation Parameters

Sr. No.	Parameter	Value
1	No. of Nodes	80
2	Area Size	500 m^2^
3	Protocol	Underwater MAC
4	Source of Traffic	CBR
5	Rate of Traffic	60 Kbps
6	Simulation Time	50 sec
7	Antenna	Omni Antenna
8	Packet Size	50, 100, 150, 200, 250 bytes
9	Channel Capacity	2 Mbps

The entire result figures show the performance comparison of IICC, MARPCP and IDACB. By this experimental analysis we found that IICC shows better results in comparison to MARPCP and IDACB because of improved data collection methodology embedding with scheduling.

**FIGURE 4.**
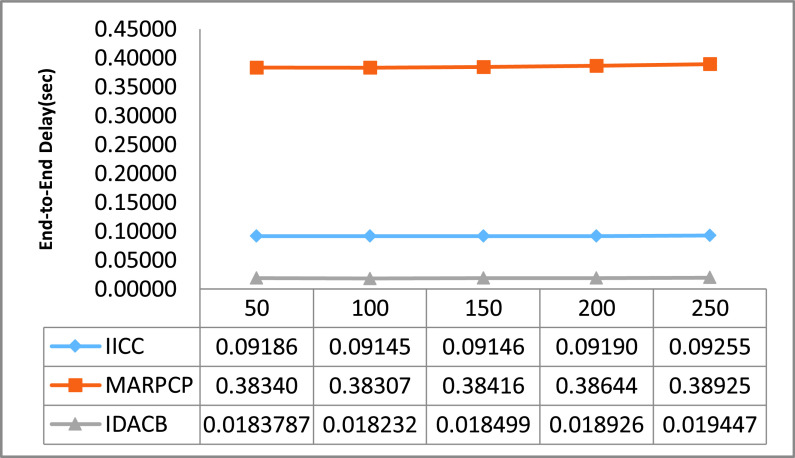
End to end delay comparison w.r.t. changing packet size.

As presented in figure 4 the comparison of end-to-end delay w.r.t. changing packet size from 50 to 250. Here as shown by the readings in this graph that the delay of MARPCP is found to be 95% lower than IDACB and 79% lower in case of IICC. Whenever, the no. of intermediate hop counts is more, then the packet time to reach the destination gets increased and leads to higher delay with higher packet delivery failure too. Due to this number of packets retransmission also gets increased and delay increases. Also with the increase packet size IICC faces higher transmission delay.

**FIGURE 5.**
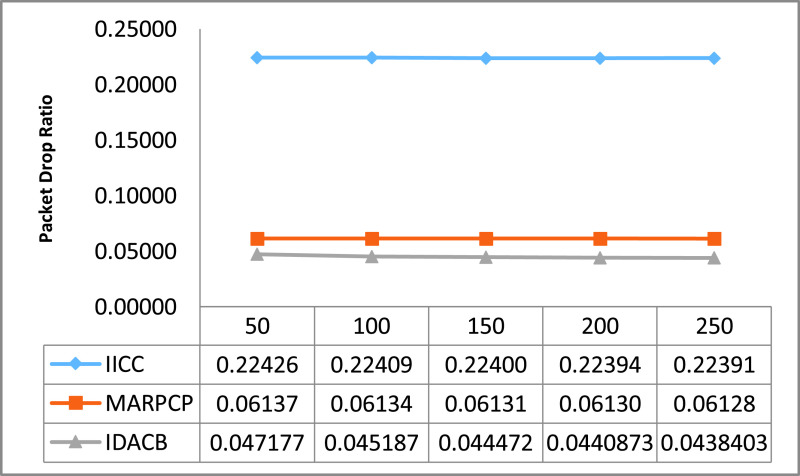
Comparison of packet drop ratio w.r.t. changing packet size.

As shown in figure 5 the comparison of packet drop ratio w.r.t. increasing continuous packet size. Here it depicts that the packet drop of MARPCP is 26% more in comparison to IDACB and 80% more in case of IICC. Whenever data packet length increases, data values sent alongside also increases for all these schemes considered here for comparison consideration. This increase in packet length can result in more errors but it is used with sleep-wake up data transmission scheduling which results in less packet drop. That’s’ why the number of retransmissions in IDACB is very less in comparison to MARPCP and IICC.

**FIGURE 6.**
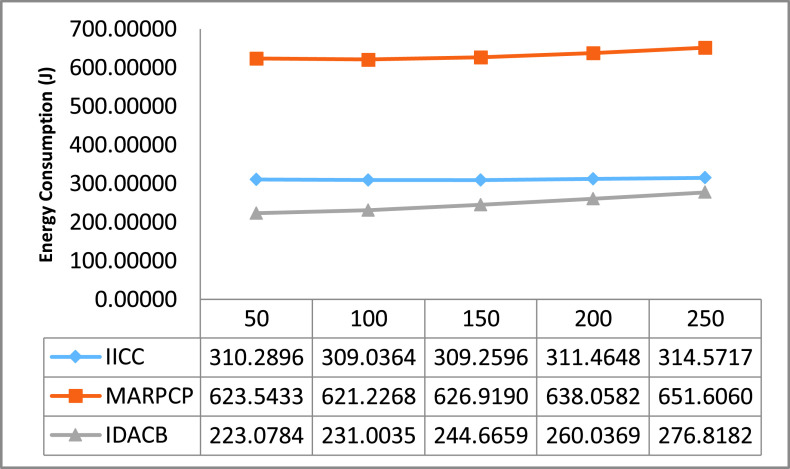
Energy consumption comparison w.r.t. changing packet size.

Further, as depicted in figure 6 the comparison of energy consumption. Here it is easily understood that the energy consumption of MARPCP is 61% higher and of IICC is 21% higher in comparison to IDACB. As reliability always remains the primary task to be ensured for every hop in the intermediate path that promises the data recovery and also avoid the source to destination retransmissions. IDACB scheme consumes very less energy that helps to increase the network lifetime.

## VI. Future Challenges and Open Issues

The various open research future challenges and issues of data collection in UAN or UWSN came into existence are as below:

1. The data collection schemes must not have overloading and congestion; otherwise data will not get collected in real-time.
2. The data collection schemes must be true and trust worthy.
3. While constructing an algorithm effective depth adjustment procedure is a challenge
4. Data collection schemes should be enough flexible to manage a fault and also during data delivery it must handle all types of faults.
5. It should confirm lowest overhead and latency.
6. For recovering burst loses, suitable loss recovery techniques should be invented during data collection.
7. The schemes of data collection should be reliable and efficient enough such that it can be implemented without any disinclination.

## VII. Conclusion

With the harsh underwater conditions of the oceans it will become the difficult task for the researchers to collect data from the nodes. UWSN is deployed for a secure and reliable collection of data. This network organization helps to discover major resources in the water by information gathering. From the above discussion it can be analyzed that data collection in UWSN is really a challenging task. The process of data collection along with classification of existing techniques is also represented here. In this manuscript, authors have discussed various existing techniques to efficiently collect data from the sink nodes. A comparative analysis of various data collection schemes w.r.t. various QoS parameters such as delay, packet drop ratio, energy consumption during communication are presented. Authors have also provided various challenges faced in data collection from UWSN and the possible approaches required overcoming these challenges. Also, authors tried to discuss some facts about existence of CORONA below the water along with water purification.
